# BIOCHEMICAL MARKERS FOR STEATOSIS: A NECESSITY. BUT FOR NOW, BE SATISFIED WITH THE BIOPSY

**DOI:** 10.1590/0102-672020230002e1721

**Published:** 2023-03-20

**Authors:** Eduardo Neubarth Trindade, Lucas dos Santos Difante, Manoel Roberto Maciel Trindade

**Affiliations:** 1 Hospital de Clínicas de Porto Alegre, Bariatric Surgery Unit, Digestive Surgery Service – Porto Alegre (RS), Brazil.

Nowadays, non-alcoholic fatty liver disease (NAFLD) is considered one of the most important causes of chronic liver disease in the world. Recent data show a global prevalence of 25%, with a recent increase that follows the current trends of the obesity epidemic^13^. Among obese patients, the prevalence of NAFLD can exceed 90%^4,10^. NAFLD is a spectrum of disease involving simple hepatic steatosis and nonalcoholic steatosis (NASH) with or without fibrosis or cirrhosis.

Regarding the disease’s natural history, about 20% of patients with hepatic steatosis have NASH. Over time, 20% of those individuals will develop hepatic cirrhosis. Recently, NASH had become the most important indication of liver transplant among women in the United States and is expected to overtake alcoholic liver disease as the leading liver transplant indication for all patients within the next few years. Compared to NAFLD, patients with NASH can develop hepatocellular carcinoma in a 12 times higher rate and have an annual mortality 1.7 times higher^9^.

Liver biopsy is the only method to diagnose NASH. It is histologic demonstrations of hepatic steatosis associated with hepatocyte ballooning degeneration and lobular inflammation. There are some clinical scores for prediction of liver fibrosis; however, these methods were not validated for the obese population and present variable specificity and sensibility in the literature^3,10,11^.

De Carli et al.^3^ evaluated the performance of these noninvasive scores in morbidly obese patients undergoing bariatric surgery. They found that the APRI (AST to platelet ration index) presented the higher specificity (99.61%), predictive positive value (PPV) (85.71%), positive likelihood ratio (PLR) (62.5), and accuracy (0.93). FIB4 was the second test in terms of accuracy (0.9) and PLR (10.53). Udelsman et al.^11^, in contrast, evaluated liver biopsies of 2465 obese patients and reported that the NAFLD fibrosis score (NFS) performed best in screening out advanced fibrosis, with a sensitivity of 85% and an NPV of 99%.

In that regard, the American Society of Bariatric and Metabolic Surgery (ASBMS) recently proposed an algorithm for screening for advanced liver fibrosis in patients seeking bariatric surgery^8^. According to the authors, it is possible to rule out advanced fibrosis in one-third of the patients undergoing bariatric surgery. In other words, if the score is under the cutoff value of -1.455, they do not to recommend performing a liver biopsy intraoperatively. In patients with indeterminate or high NFS, liver stiffness measure or intraoperative liver biopsy should be considered.

However, we must emphasize that NAFLD is a variable spectrum of disease, which reduces the accuracy of clinical scores and makes it difficult to identify specific markers for screening for the disease. The condition also has a major burden in the public health system due to its natural history and negative outcomes. Therefore, in the context of bariatric surgery, it is very important to screen the patients for advanced fibrosis and cirrhosis, since it may alter the follow-up and treatment after surgery. Intraoperative liver biopsy is relatively safe because it is possible to observe and confirm hemostasis, unlike percutaneous needle biopsy^12^. We also believe that the additional operative time (around 5 min) and the cost of processing the specimen do not overcome the potential benefits of an accurate diagnosis.

There are no NASH-specific medications. Weight loss is the first treatment goal if it has the strongest association with histologic improvement^1,2^. Currently, bariatric surgery is the most effective weight-loss therapy, which plays a crucial role in the management of obese patients with NAFLD and NASH. Studies comparing liver biopsies before and after bariatric surgery have shown substantial improvements in liver histology, including fibrosis regression^5–7^.

The NAFLD inflicts a great burden on patients’ and on the health system. As long as we do not find an accurate biochemical marker or clinical score, intraoperative liver biopsy remains the best alternative for an adequate diagnosis in patients undergoing bariatric surgery. It allows the proper follow-up and treatment after the surgery. Routine hepatic biopsy during surgery makes possible to both the physician and the patient to know the real extent of the problem and manage it properly. The high prevalence of NAFLD in this population overcomes any possible complications or extra cost of the procedure.
